# The effects of magnetic fields exposure on relative permittivity of saline solutions measured by a high resolution SPR system

**DOI:** 10.1038/srep25111

**Published:** 2016-04-28

**Authors:** Li Jiang, Xinyuan Zhao, Yue Fei, Dongdong Yu, Jun Qian, Jinguang Tong, Guangdi Chen, Sailing He

**Affiliations:** 1 State Key Laboratory of Modern Optical Instrumentation (Zhejiang University), Centre for Optical and Electromagnetics Research, Zhejiang Provincial Key Laboratory for Sensing Technologies, JORCEP (Sino-Swedish Joint Research Center of Photonics), Zhejiang University, Hangzhou 310058, China; 2 Bioelectromagnetics Laboratory, Zhejiang University School of Medicine, Hangzhou 310058, China

## Abstract

A measurement system for the relative permittivity of a physiological solution under 50 Hz magnetic fields (MF) is presented. It is based on a phase-sensitive surface plasmon resonance (SPR) system. Relative permittivity was analyzed for different solute concentrations of sodium chloride under various MF exposure parameters. We found that MF exposure at 0.2–4.0 mT step-wise decreased significantly the SPR phase signal of a 0.9% sodium chloride solution while 0.1 mT of MF exposure did not. The decreases in the SPR phase signal depended on the duration of MF exposure, and the signal reached a plateau after 15 min of exposure. Interestingly, the decreased SPR phase signal showed a gradual increase and approached the background level when the exposure was drawn off. In addition, we found that the response of the sodium chloride solution to MF also depended on its concentration. In brief, the relative permittivity of sodium chloride in solutions appears to be practically affected by 50 Hz MF exposure. Our data indicates that the relative permittivity of the saline solution influenced by MF exposure should be considered when investigating the biological effects of MF exposure on organisms in experimental study.

With the development of the electric power industry and wide usage of electronic products, the public concerns have been raised on exposure to increasing electromagnetic fields, especially extremely low frequency magnetic fields (ELF-MF), which range from 1–300 Hz. In 2002, the International Agency for Research on Cancer (IARC) has classified ELF-MF as a possible carcinogen to humans according to limited epidemiological evidences^1^. It remains inconclusive whether exposure to ELF-MF increases risks of other detrimental health effects^2^. Moreover, laboratory studies did not clarify the biological effects induced by ELF-MF and their underlying mechanisms^3^,^4^,^5^. The critical issue is that the biophysical mechanisms of ELF-MF acting on organisms are not clear.

Dielectric properties are vital biophysical features of biological tissues, and biological activity is an index to assess the active state of tissues. Wang *et al*. has reported that the dielectric properties of human tissue are related to its biological activities^6^. The permittivity, which is a vital dielectric property related to the ability to store electric charge, has a great value in various medical areas including electromagnetic protection, electromagnetic imaging, and disease diagnosis^7^,^8^,^9^. Thus, it is reasonable that the normal dielectric properties of tissues or cells are important for maintaining their biological function. It is well known that permittivity is related to refractive index. For the saline solution, a non-magnetic material, μ_r_ is very close to 1, thus, we can obtain 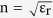, where ε_r_ represents the relative permittivity and n represents the refractive index^10^,^11^. To that end, we studied the effects of ELF-MF exposure on refractive index with phase-interrogation surface plasmon resonance (SPR) sensing.

Surface plasmons are free electron oscillations at the metal-dielectric interface and can be optically excited^12^. SPR sensing is an optical refractive index sensing technology with high sensitivity, label-free and real-time detection^13^. It has been widely applied in many areas, including proteomics, pharmacy, genomics, pollutants detection, and food safety^14^,^15^,^16^,^17^,^18^,^19^,^20^,^21^. Conventional SPR sensors are based on amplitude detection and can be classified as intensity-interrogation^22^, angle-interrogation^23^ and wavelength-interrogation^24^. SPR sensors based on phase-interrogation have been extensively studied since the late 1990s, because their resolution is 2 to 3 orders of magnitude higher than that of amplitude-interrogation (10^−7^–10^−8^ RIU)^10^,^25^,^26^,^27^. The change in permittivity of the sample could be reflected by the SPR phase signal change. That means, when the change in SPR phase signal is detected, we can consider that the permittivity of the sample changes. In this study, we have obtained the SPR phase signals of sodium chloride solutions under ELF-MF exposure with various parameters in a phase-interrogation SPR sensing system based on a novel parallel-moving prism phase modulator (PM-PPM). The effects of ELF-MF on sodium chloride solutions with different concentrations have been studied by analyzing the changes in SPR phase signals.

## Results

**Figure 1:**
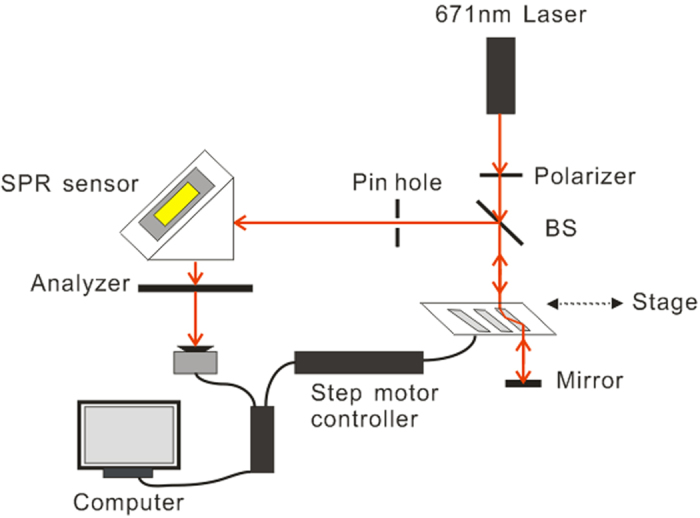
Schematic diagram showing the setup of phase-sensitive SPR sensing system based on PM-PPM.

Figure 1 shows the setup of our phase-sensitive SPR sensing system. In this system, a stabilized laser with a wavelength of 671 nm was used as the light source. The light went through a linear polarizer and a beam splitter, and was then incident into the PM-PPM, which was equipped with three BK7 rhombic prisms. A mirror was used to reflect the light back into the prism. The modulated light was then filtered with a pin hole to reduce the stray light and struck the SPR sensor at an appropriate angle to meet the resonance condition. The reflected light from the SPR sensor passed through an analyzer, and then the interference light was detected by a photo detector. The SPR sensor in the Kretschmann configuration included a homemade microfluidic chip (size: 25.4 × 25.4 mm^2^) with one reaction chamber and one gold spot, and a right angle coupling prism.

### Detection Sensitivity

**Figure 2:**
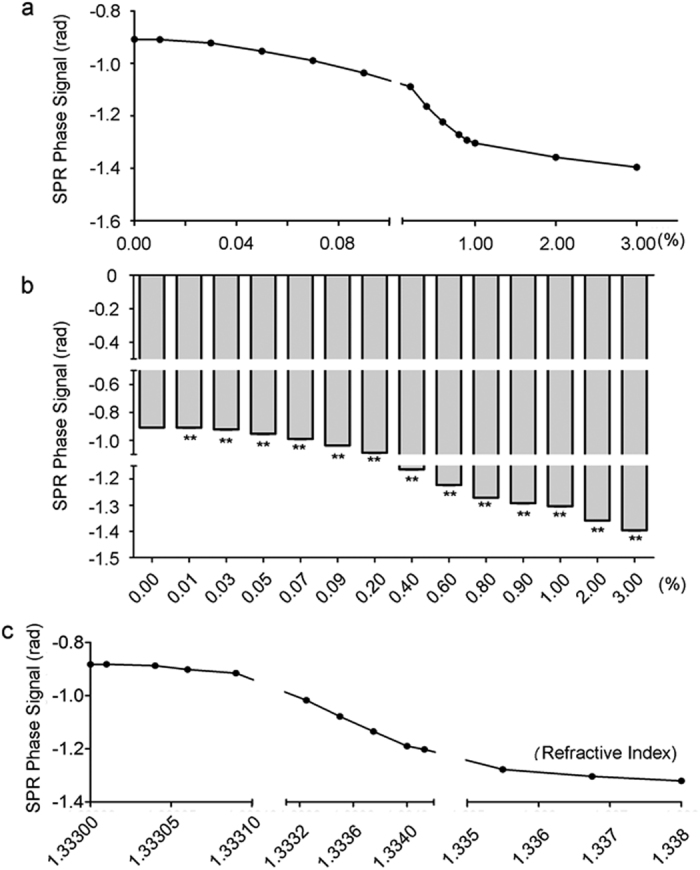
SPR phase signals of sodium chloride solutions with different concentrations. (**a**) Representative SPR phase signal curve of different concentrations of sodium chloride solutions. (**b**) A graph showing a statistical analysis of the SPR phase signal in different concentrations of sodium chloride solutions compared with 0.00%. All the quantitative data are presented as mean ± SD, **P < 0.01. (**c**) SPR phase signals corresponding to glycerin solutions with different refractive index values known at the wavelength of 671 nm.

To explore the detection sensitivity of SPR on the refractive index, the SPR signals of different concentrations of sodium chloride solutions were measured at a constant temperature of 25 °C, since it is well known that the refractive index of chloride solution is positively related with concentration. The results showed that the SPR signal was gradually increased following increased concentration (Fig. 2a,b). We also found that the detection limit on the concentration of SPR was lower than 0.01%, suggesting a relatively high sensitivity. SPR phase corresponding to glycerin solutions with various known refractive index values were also detected (Fig. 2c). From this we could see that the sensitivity and resolution of our system were about 1900 rad/RIU and 3.421 × 10^−7^ RIU (calculated using Equ. (S1) and (S2)). The curve between every two spots in Fig. 2(c) could be seen as a linear line, which could be used to estimate refractive index change of sodium chloride solutions after MF exposure (Supplementary: Table S1).

### Study on the Effects of ELF-MF on the Permittivity of Sodium Chloride Solutions

**Figure 3:**
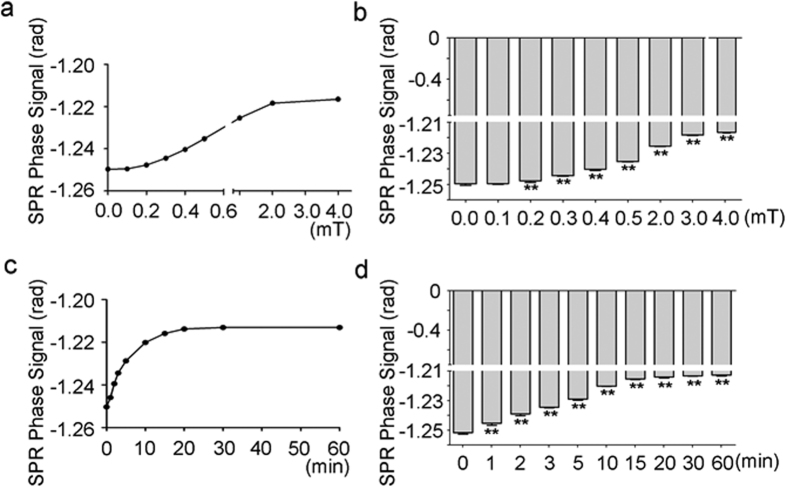
The effects of MF exposure on SPR signals of sodium chloride solutions. (**a**) MF decreased the SPR signal in a time-dependent manner. (**b**) A graph showing a statistical analysis for (**a**). (**c**) MF decreased the SPR signal in a dose-dependent manner. (**d**) A graph showing a statistical analysis for (**c**). All the quantitative data are presented as mean ± SD, **P < 0.01 (comparing to the control of shame exposure).

**Figure 4:**
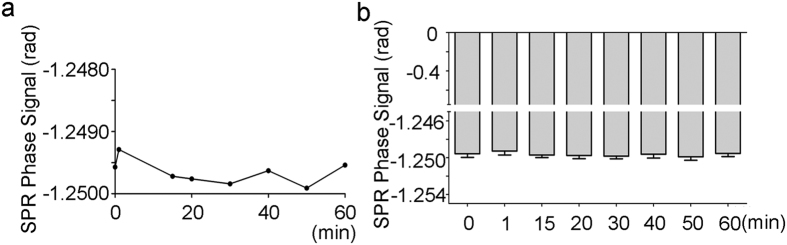
The effects of MF exposure at 0.1 mT on SPR signals in 0.9% sodium chloride solutions. (**a**) The effects of 0.1 mT MF on the SPR signals of the solution for the indicated time. (**b**) A graph showing a statistical analysis for (**a**) (compared to the control of shame exposure).

By exploring the effects of ELF-MF on the relative permittivity of sodium chloride solutions, an SPR signal of 0.9% sodium chloride solution, a physiological saline solution, was measured in the presence or absence of 50 Hz MF exposure. The results demonstrated that the SPR phase signal decreased under MF exposure in a time-dependent manner when compared to the control experiment, which did not have MF exposure (Fig. 3a,b). Interestingly, the SPR phase signal reached a constant value after 15 min of MF exposure. Next, we exposed sodium chloride solutions to different exposure intensities of MF for 15 min and measured the SPR signals. We found that MF decreased SPR signals in a dose-dependent manner, and MF at as low as 0.2 mT could significantly increase the SPR signal. However, 0.1 mT of MF exposure did not show any significant change (Fig. 3c,d). To evaluate whether MF at 0.1 mT affect the SPR signal for longer exposure durations, we exposed the solution to 0.1 mT MF for a longer time, and found that none of the indicated exposure durations successfully affected the signal, indicating that 50 MF with an exposure intensity higher than 0.1 mT is necessary for initiating a response from the SPR signals (Fig. 4a,b). Taken together, these results suggested that MF exposure decreases the permittivity of sodium chloride solutions in a time and dose dependent way. One spot (50 Hz, 4.0 mT, 15 min) of 0.9% sodium chloride solution was chosen to estimate the refractive index change caused by MF exposure. According to the curve between SPR phase signal and refractive index (Fig. 3c), the change was about −3.95 × 10^−4^ RIU (Supplementary: Table S1).

### The Effects of the ELF-MF on Permittivity of Sodium Chloride Solutions is Reversible

**Figure 5:**
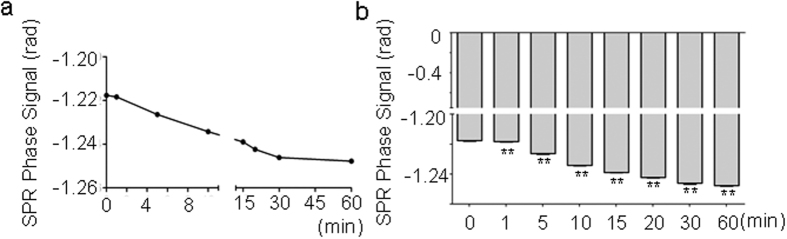
Recovery of SPR signals of sodium chloride solutions when the MF exposure was drawn off. (**a**) SPR signal (that has been decreased by MF earlier) now increases (i.e., recover) in a time-dependent way. (**b**) A graph showing a statistical analysis for (**a**). All the quantitative date are presented as mean ± SD, **P < 0.01 (comparing to the control of shame exposure).

We next evaluated if the effects of MF exposure on SPR signals in sodium chloride solutions were reversible. To that end, we exposed the solution to MF at 4.0 mT for 15 min and then had the exposure drawn off. We found that the MF-decreased SPR phase signals began to intensify immediately even only 1 minute after exposure, gradually increased during the recovery time, and almost approached the background level (before any MF exposure) after 60 min (Fig. 5a,b). These results indicate that permittivity decreased by MF is reversible.

### The Effects of MF on Permittivity is Dependent on Concentration of Solutions

**Figure 6:**
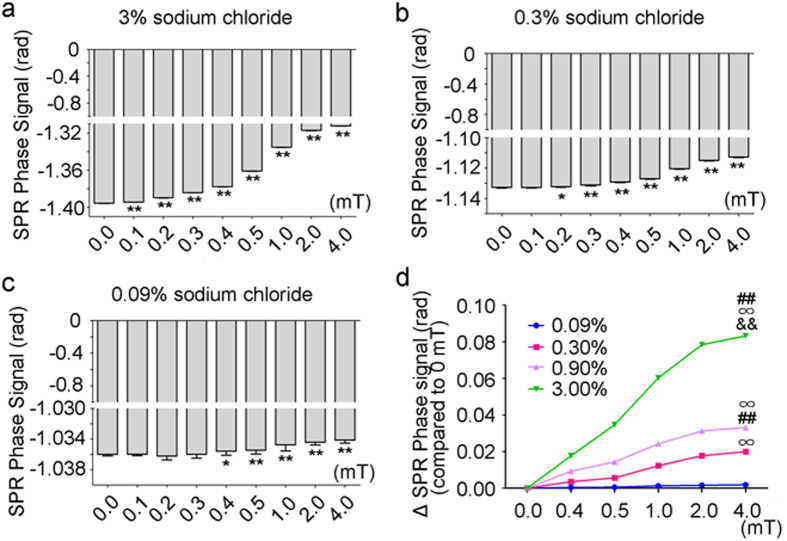
The effects of MF exposure on SPR signals of sodium chloride solutions with different concentrations. (**a**) The effects of MF of various magnitudes on a SPR signal of 3% sodium chloride solutions. (**b**) The effects of MF on a SPR signal of 0.3% sodium chloride solutions. (**c**) The effects of MF on a SPR signal of 0.09% sodium chloride solutions. (**d**) SPR signal responses of sodium chloride solutions with different concentrations as the magnitude of the MF exposure increases. All the quantitative data are presented as mean ± SD, *P < 0.05, **P < 0.01.

Since the decreases of SPR phase signals of sodium chloride solutions depend on MF exposure duration and dose, we next evaluated whether these effects depend on the solution itself, e.g., the concentrations of the solution. The SPR signals of sodium chloride solutions at 0.09%, 0.3% 0.9% and 3% were examined. The results showed that MF exposure to 0.1 mT for 15 min was able to significantly affect the SPR signals of 3% sodium chloride solutions but not lower concentrations of solutions (Fig. 6a–c). The MF exposure threshold for a SPR signal response of the 0.09% solution was up to 0.4 mT, suggesting that a lower solution concentration of sodium chloride results in a higher responsive threshold of MF exposure (Fig. 6c). In addition, we found that the solution concentration was a critical factor for the amplitude of the decreased SPR signals. The solutions with higher concentration were more responsive to MF under the same exposure parameters (Fig. 6d). Interestingly, we found similar response of human serum to ELF-MF exposure, in which the 50 Hz at 4.0 mT could decrease the amplitude of SPR phase signal (Supplementary: Fig. S1). These results indicate that bodily fluids for organisms might respond to MF differently.

## Discussion

In this study we have measured relative permittivities (refractive index) of sodium chloride solutions under 50 Hz magnetic fields based on a phase-sensitive surface plasmon resonance (SPR) system with a high sensitivity of about 1900 rad/RIU and a high resolution of about 3.421 × 10^−7^ RIU. We have found that SPR phase signals (monotonously related to the relative permittivity) decrease under MF exposure in a time and dose dependent manner, and the decreased SPR phase signals were reversible when the exposure was drawn off. Furthermore, the SPR signal responses of sodium chloride solutions depend on the solute concentrations. For 0.9% sodium chloride solution, the refractive index decreased by 3.95 × 10^−4^ RIU after MF exposure (50 Hz, 4.0 mT, 15 min). Similar to 50 Hz MF exposure, we found that exposure to ELF-MF of other frequency (e.g., 30 Hz and 130 Hz) could decrease SPR phase signals in sodium chloride solutions (Supplementary: Fig. S2). Abbe refractometer was also used to measure the refractive index of 0.9% sodium chloride solution after MF exposure (Supplementary: Fig. S3), which was in good agreement with the results of SPR sensing. However, compared to abbe refractometer, SPR sensing offers higher sensitivity and resolution, which can detect much smaller refractive index change. Our data suggest that the relative permittivity of a saline solution is influenced by MF exposure, which should be considered in experimental studies on the biological effects induced by MF exposure.

It has been reported recently that SPR sensing can be enhanced based on nanomaterials which can modify the sensing substrate or be used as an amplification tag, which can enhance the electric field of the sensing substrate^28^. With the improvement of the sensitivity, phase-sensitive SPR sensing can be used to monitor the interaction between proteins^19^ and detect specific molecules with extremely low concentration^29^.

## Methods

### Theoretical Analysis

The Kretschmann configuration is used to optically excite SPR in our phase-sensitive SPR system. In the Kretschmann configuration, a 3 nm chromium layer and a 50 nm gold layer are deposited on a BK7 glass slide in sequence, which couples to the bevel of a coupling prism. When total internal reflection (TIR) occurs on the prism/gold interface, an evanescent wave is induced with the wave vector parallel to the interface as


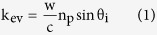


where n_p_ represents the refractive index of the coupling prism, θ_i_ is the incident angle on the prism/gold interface, wis the angular frequency of the incident light, and c is the speed of light. From Maxwell’s equations, the propagation constant of the surface plasmons confined to the interface between the gold and the sample can be deduced as


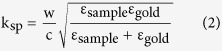


where ε_sample_ and ε_gold_ are the dielectric constants of the sample and the gold, respectively.

When k_ev_ = k_sp_, the surface plasmon can be resonantly excited. The reflected p-polarized light suffers a phase jump while the phase of the s-polarized light stays almost unchanged. As a result, a phase difference φ_spr_ is created, which is related to the refractive index of the sample in contact with the gold surface. In our system, a parallel-moving prism phase modulator (PM-PPM) is used to extract φ_spr_. In the novel proposed PM-PPM, the phase difference between p-polarized and s-polarized light can be modulated by the TIR in an uncoated rhombic prism. It is apparent to all that TIR occurs on a prism/air interface when the light incident angle θ_i_ is larger than the critical angle, which will lead to a different phase shift of p-polarized light and s-polarized light. From the formula of the reflectivity of p-polarized and s-polarized light (i.e., the Fresnel formula), the phase difference φ_m_ after m times TIR can be calculated as follows^30^,^31^,


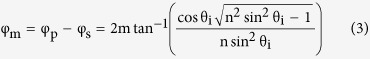


where n represents the refractive index of the prism. φ_p_ and φ_s_ are defined as the phase shifts of p-polarized light and s-polarized light after m times (m counts) of TIR at the incident angle of θ_i_. It is worth noting that φ_m_ is determined by the refractive index of the prism, the incident angle and the TIR count. When the refractive index of the prism and the TIR count are fixed, we can achieve different phase difference between p-polarized and s-polarized light by modulating the incident angle. The rhombic prism is quite suitable to be used as a phase difference modulator in phase-sensitive SPR biosensors because of the unique characteristics of the TIR.

The interference intensity between the projection of p-polarized and s-polarized can be described as,


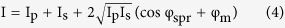


where I_p_ and I_s_ are the projection intensities of the reflected p-polarized and s-polarized light on the axis of the linear analyzer. By the expansion of the cosine component, the interference intensity can also be written as,


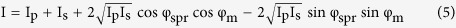


When I_A_ = I_p_ + I_s_, 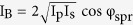 and 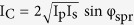, the interference intensity can be simplified to


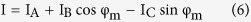


The phase of SPR can be extracted by


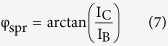


Three different φ_mi_(i = 1, 2, 3), i.e., φ_m1_ = 0.85π, φ_m2_ = 0.66π and φ_m3_ = 0.40π can be offered by PM-PPM with three prisms fixed on a motor-driven stage in different incident angles, and the related I_i_ (i = 1, 2, 3) can be detected correspondingly. The interference intensity can be written in the form


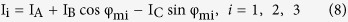


As a result, the phase of SPR φ_spr_ can be extracted after I_B_ and I_C_ are calculated.

### MF Exposure System

The ELF-MF exposure system (OX9-1) used in this study was designed and provided by Dr. Bao^32^. It consists of a “U” type magnet enwrapped in coils, a current amplifier, and a central regulator. Once initiated, ELF-MF (e.g., 50 Hz MF) was produced inside the magnet, and the central regulator was used to control the intensity and frequency. The control group was conducted under the same conditions but without the ELF-MF exposure.

### Statistical Analysis

For each experiment, the value was presented as means ± standard deviation (SD). All computations were performed with SPSS 16.0. The statistical differences between exposed and sham-exposed were tested using the Dunnett’s two-tailed *t*-test. A probability level of *P* < 0.05 was considered statistically significant.

## Additional Information

**How to cite this article**: Jiang, L. *et al*. The effects of magnetic fields exposure on relative permittivity of saline solutions measured by a high resolution SPR system. *Sci. Rep*. **6**, 25111; doi: 10.1038/srep25111 (2016).

## Supplementary Material

Supplementary Information
