# Supplementary material for: The effects of magnetic fields exposure on relative permittivity of saline solutions measured by a high resolution SPR system
Source: Sci Rep. 2016 Apr 28;6:25111. doi: 10.1038/srep25111 (PMC4848526; doi:10.1038/srep25111)
Supplement: Supplementary Information [file srep25111-s1.pdf]

Supplementary Information

The effects of magnetic fields exposure on relative permittivity of saline solutions measured by a high resolution SPR system

Li Jiang[1#], Xinyuan Zhao[2#], Yue Fei[2], Dongdong Yu[2], Jun Qian[1],Jinguang Tong[1], Guangdi Chen[2*], Sailing He[1*]

[1] State Key Laboratory of Modern Optical Instrumentation (Zhejiang University), Centre for Optical and Electromagnetics Research, Zhejiang Provincial Key Laboratory for Sensing Technologies, JORCEP (Sino-Swedish Joint Research Center of Photonics), Zhejiang University, Hangzhou 310058, China.

[2] Bioelectromagnetics Laboratory, Zhejiang University School of Medicine, Hangzhou 310058, China.

Results

Detection Sensitivity and Resolution

Fig. 2(c) shows the SPR phase signals of glycerin solutions with different refractive index values. The SPR phase signals from 1.33300 to 1.338 were -0.882rad, -0.8819rad, -0.8874rad, -0.9019rad, -0.9151rad, -0.9531rad, -1.0172rad, -1.0781rad,-1.1346rad, -1.1896rad, -1.202rad, -1.214rad, -1.2777rad, -1.3039rad and -1.3208rad, respectively. The region when the glycerin refractive index value increased from 1.33309 to 1.33311 with the phase signal noise of 0.00065rad was selected to calculated the phase sensitivity and resolution.

$$S_p = \frac{\Delta\varphi_{spr}}{\Delta n} = 1900 rad/RIU \quad \text{(S1)}$$

$$\mathrm{R} = \frac{\sigma}{S_p} = 3.421 \times 10^{-7} RIU \quad \text{(S2)}$$

Where $\Delta\varphi_{spr}$ and Δn are the change of the SPR phase signal and refractive index value, S_p and R are the phase sensitivity and resolution of the system, respectively.

Study on the Effects of ELF-MF on the Permittivity of Human Serum

Our results showed that human serums from five individual persons could be affected by the MF exposure (50 Hz, 4.0 mT, 15 min) which could cause the amplitude of SPR phase signal decreased (Fig. S1(a)). Then, we exposed the human serum from the second person to various exposure intensities of MF for 30min and measured the SPR phase signal (Fig. S1(b, c)). The result showed that MF exposure could also affect the human serum in a dose dependent way, which was similar to the result of 0.9% sodium chloride solution.

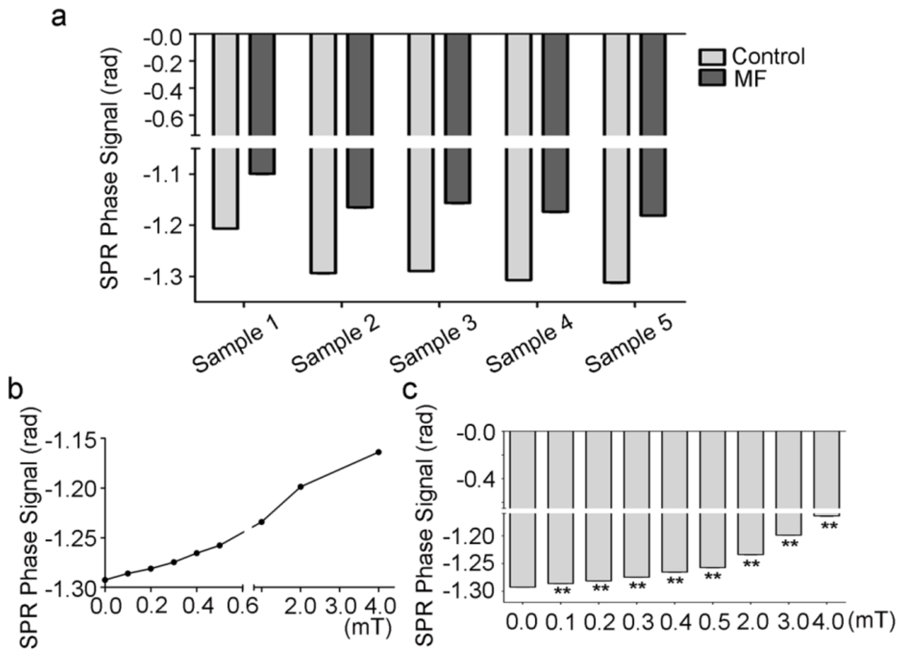

Figure S1. The effects of MF exposure (50 Hz, 4.0 mT, 30 min) on SPR signals of human serum. (**a**) The effects of MF exposure on a SPR signal of five individual persons (sample 1, 2, 3, 4, 5). (**b**) MF decreased the SPR signal of one person (sample 2) in a dose-dependent manner. (**c**) A graph showing a statistical analysis for Fig. S1 (b) All the quantitative data are presented as mean±SD, **P<0.01 (comparing to the control of sham exposure).

Study on the Effects of ELF-MF on the Permittivity of 0.9%Sodium Chloride Solution

The SPR phase signals of 0.9% sodium chloride solution under (30 Hz, 2.0 mT, 15 min) MF exposure and (130 Hz, 2.0 mT, 15 min) MF exposure were decreased. Furthermore, MF exposure could affect the 0.9% sodium chloride solution in a frequency dependent way. The amplitude of SPR phase signal decreased with the increasing of MF exposure frequency (Fig. S2).

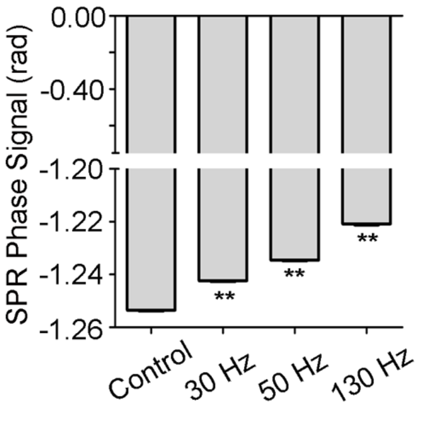

Figure S2. MF exposure (2.0 mT, 15 min) decreased the SPR signal of 0.9% sodium chloride solution in a frequency-dependent manner.

Measure the Refractive Index of 0.9%Sodium Chloride Solution Based on Abbe Refractometer

We also used an abbe refractometer (2WA-J) to detect the refractive index of 0.9% sodium chloride solution under ELF-MF exposure. The refractive index of ten groups of 0.9% sodium chloride solution after (50 Hz, 4.0 mT, 15 min) MF exposure and ten sham groups were detected. The result with statistical evaluation showed that MF exposure could cause the refractive index decrease (Fig. S3), which was agreement with the result that the refractive index decreased by 3.95×10^{-4} RIU detected by the SPR sensing.

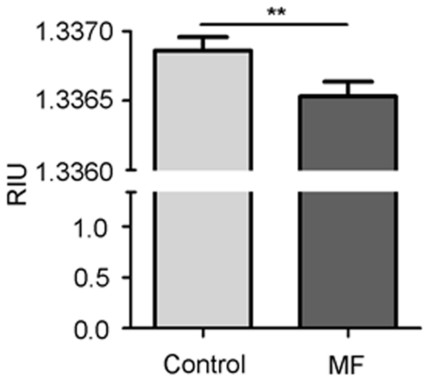

Figure S3. A graph showing a statistical analysis on refractive index of 0.9% sodium chloride solution measured by abbe refractometer.

The Refractive Index Change Caused by MF Exposure

From Fig. 2(c), the curve between every two spots could be seen as a linear line. We could obtain the linear relationship as shown in Table 1. One spot (50 Hz, 4.0 mT, 15 min) of 0.9% sodium chloride solution was chosen to estimate the refractive index change caused by MF exposure. The SPR phase of 0.9% sodium chloride solution after MF exposure (50 Hz, 4.0 mT, 15min) was -1.2154rad. From Table S1, the linear relationship $\varphi_{spr} = -50.96n + 66.779$ could be chosen to calculate the corresponding refractive index of 0.9% sodium chloride solution. The calculated refractive index was 1.33427 RIU. Compared to the refractive index (1.334665 RIU) of 0.9% sodium chloride solution under no MF exposure, the refractive index changed by -3.95×10^{-4} RIU.

$\varphi_{spr}(rad)$	$\varphi_{spr}(rad) - \text{n(RIU)}$
[-0.8874, -0.8820]	$\varphi_{spr} = -183.333n + 243.503$
[-0.9019, -0.8874]	$\varphi_{spr} = -725n + 965.566$
[-0.9151, -0.9019]	$\varphi_{spr} = -440n + 585.644$
[-0.9531, -0.9151]	$\varphi_{spr} = -1900n + 2531.956$
[-1.0172, -0.9531]	$\varphi_{spr} = -457.857n + 609.421$
[-1.0781, -1.0172]	$\varphi_{spr} = -243.6n + 323.762$
[-1.1896, -1.0781]	$\varphi_{spr} = -223n + 296.292$
[-1.2020, -1.1896]	$\varphi_{spr} = -95.384n + 126.053$
[-1.2140, -1.2020]	$\varphi_{spr} = -100n + 132.211$
[-1.2777, -1.2140]	$\varphi_{spr} = -50.96n + 66.779$
[-1.3039, -1.2777]	$\varphi_{spr} = -20.96n + 26.714$
[-1.3208, -1.3039]	$\varphi_{spr} = -13.52n + 16.769$

Supplementary Table S1. The linear relationship between SPR phase signal and refractive index in every two spots in Fig. 2(c).

Data Processing

The interference intensity can be written as equation (6).

Three rhombic prisms are mounted on the motor-driven stage in different incident angles. As a result, three different modulated phases can be obtained, which represent the phase difference between p-polarized light and s-polarized light. A LabVIEW program is developed to manipulate the moving of the motor-driven and to save the acquisition data from the detector, which can move the first rhombic prism into the light path and then save the interference intensity. That means, as for one sample, one rhombic prism representing one phase modulation φ_m corresponds to one interference intensity I. Three interference intensities can be obtained by, $I_1 = I_A + I_B\cos\varphi_{m1} - I_C\sin\varphi_{m1}$, $I_2 = I_A + I_B\cos\varphi_{m2} - I_C\sin\varphi_{m2}$, $I_3 = I_A + I_B\cos\varphi_{m3} - I_C\sin\varphi_{m3}$, where $\varphi_{m1} = 0.85\pi$, $\varphi_{m2} = 0.66\pi$ and $\varphi_{m3} = 0.40\pi$ obtained according to equation (3), I_1, I_2 and I_3 are collected by the detector. As a result, I_A, I_B and I_C can be calculated by the MATLAB processing. The phase of SPR can be extracted using equation (7). Ten groups of data were saved and processed to reduce the noise. In addition, statistical analysis was given to improve the accuracy of the experiment.

All the experiments were carried out in our clean room with a constant temperature at 25 ℃, which could reduce the noise from the temperature fluctuations and ensure no temperature variation during the MF exposure. The sample was placed on the MF exposure system using a 5mL centrifuge tube and was

injected into the homemade PDMS microfluidic chip automatically by a syringe mounted on an injected pump.

Human Serum and Ethnics

Health human serum samples were collected at the time of routine heamotological examination from the department of laboratory, the Sir Run Run Shaw Hospital, Zhejiang University School of Medicine. Informed consent was obtained from each individual, and approval for the study protocol was granted by the ethical committee of the Sir Run Run Shaw Hospital, Zhejiang University School of Medicine.
